# Play in Children with Neurodevelopmental Disorders: Psychometric Properties of a Parent Report Measure ‘My Child’s Play’

**DOI:** 10.3390/children8010025

**Published:** 2021-01-06

**Authors:** Dulce Romero-Ayuso, María Ruiz-Salcedo, Sabina Barrios-Fernández, José Matías Triviño-Juárez, Donald Maciver, Janet Richmond, Miguel A. Muñoz

**Affiliations:** 1 Physical Therapy Department, Occupational Therapy, Faculty of Health Sciences, University of Granada, 18016 Granada, Spain; mariamrs97@hotmail.com; 2 Medical-Surgical Therapeutics Department, University of Extremadura, 10003 Cáceres, Spain; sabinabarrios@unex.es; 3 Primary Care Center Zaidín Sur, Granada Metropolitan Sanitary District, 18007 Granada, Spain; jmtjuarez@hotmail.com; 4 Occupational Therapy and Arts Therapies Subject Area, School of Health Sciences, Queen Margaret University, Edinburgh, Scotland EH21 6UU, UK; DMaciver@qmu.ac.uk; 5 Occupational Therapy, School of Medical and Health Sciences, Edith Cowan University, 270 Joondalup Drive, Joondalup WA 6027, Australia; inscribeot@outlook.com.au; 6 Personality, Assessment and Psychological Treatment Department, Faculty of Psychology, University of Granada, 18071 Granada, Spain; mamuoz@ugr.es

**Keywords:** play, assessment, executive functions, neurodevelopmental disorders, autism spectrum disorder, specific language disorder

## Abstract

Play is essential in childhood, allowing for a positive trend in development and learning. Health professionals need useful tools to assess it, especially in the case of children with neurodevelopmental disorders. The aim of this study was to validate and cross-culturally adapt the My Child’s Play questionnaire and to find out if this instrument allows us to differentiate the play of children with neurodevelopmental disorders from the play of children with neurotypical development. A total of 594 parents completed the questionnaire. A confirmatory factor analysis was conducted, which showed a similar structure to the English version: (1) executive functions; (2) environmental context; (3) play characteristics; and (4) play preferences and interpersonal interactions. The reliability of the analysis was high, both for the whole questionnaire and for the factors it comprises. The results provide evidence of the potential usefulness of the My Child’s Play questionnaire for determining play needs and difficulties of children; moreover, this tool can also be used to plan intervention programs according to the needs of each child and family.

## 1. Introduction

Play is considered a natural learning mechanism through which children explore and learn about themselves and the world around them [1]. Children spend considerable time playing, providing fun, learning, and activity [2]. Children’s play has been studied by many disciplines because of its considerable influence on global development and well-being, being essential for achievement of motor, cognitive, emotional, and social milestones [3]. In fact, participation in play contributes to peer inclusion, improvement of self-concept and self-esteem, promotion of creativity and flexibility, emotional regulation, language development, and frustration tolerance during academic and daily life activities [1]. Play can be defined as a non-serious, spontaneous, or organized activity that provides enjoyment, entertainment, amusement, or diversion and its characteristics include intrinsic motivation, emphasis on process rather than product, pleasure, reward, and voluntary engagement [2,4].

In children with neurodevelopmental disorders (NDD), especially those with autism spectrum disorder (ASD), developmental delay (DD), and specific language disorder (SLD), differences are observed in the way they participate in play [5,6]. In ASD diagnosed children, deficits in joint attention, imagination, imitation, and communicative intention can affect their play development [7]. Usually, their play interests are oriented toward sensory and physical aspects [8], and together with their repetitive and stereotypical behaviors [9] produce issues while interacting with their peers and achieving a proper play engagement [10,11]. Children with DD spend more time in passive activities rather than playing and with adults more than with their peers [12]. Although children with ASD and SLD diagnosis face different challenges around play, there are few studies that address them, and the instruments to assess them are limited [1,13,14].

Educational and health professionals may use different tools for play assessment [6], such as the Test of Playfulness, Test of Environmental Supportiveness, Revised Know Preschool Play Scale, The McDonald Play Inventory, The Play Assessment for Group Settings, and My Children’s Play. Test of Playfulness [15] is designed to measure play in children between 6 months and 18 years, observing their individual free play both indoors and outdoors and its extension. The Test of Environmental Supportiveness assesses how the environment influences play. The Revised Knox Preschool Play Scale [16] uses observation to assess participation in play, taking into consideration the use of space and materials, sensory and motor processing, social behavior, communication, and symbolic play. The McDonald Play Inventory [17] is a two-part self-report instrument that provides information about play activities and play style. For the pretend play evaluation, the Play Assessment for Group Settings [18] and the Child-Initiated Pretend Play Assessment [19,20] are available, having this second one an extension for measuring social aspects of play called the Indigenous Play Partner Scale, both using professional observation.

On the other hand, as parents are often the primary caregivers of their children, it is important to know the way in which they interact during play situations [21]. It is also important to consider parents’ beliefs about their children’s play, influencing how they organize their contexts, activities, and interactions [1,22,23]. Although parents are able to adapt their play to their child’s play level, parents with ASD children can use fewer symbolic solicitations, not giving the opportunity to make their play more complex [24]. For all the above, parents must be considered as important agents for a proper play assessment and intervention.

The My Children’s Play (MCP) questionnaire [25] is a measure that provides information about parent’s perception about their child’s play performance, where they indicate the response that best describes their child’s play behavior on a 5-point Likert scale. This instrument has a total of 43 items divided in four factors: executive functions, interpersonal relationships, play choices, and preferences and opportunities in the environment, which makes it a tool with a broad view on a phenomenon as complex as children’s play.

The availability of valid, reliable, and appropriate tools to assess play using parental perceptions and beliefs is important for a complete understanding of children’s play. The first aim of this study was to conduct a cross-cultural adaptation of the My Child’s Play questionnaire for the Spanish-speaking population. Secondly, we studied whether the My Child’s Play questionnaire would allow us to know the characteristics of the play of children with NDD, such as ASD, DD, and SLD, and to differentiate them from children with neurotypical development.

## 2. Materials and Methods

### 2.1. Participants

The participants were recruited through different educational centers and associations of children with functional diversity: CEIP Parque de la Infantas de Granada, CEIP Maruja Mallo de Málaga, Asociación Serranía de Churriana, Federación Española de Autismo (FESPAU), Autismo España, and Asperger España. Two researchers with experience in child therapy contacted the different entities (M.R.-S. and D.R.-A.) A researcher with clinical experience in children with ASD and SLD recruited the parents of children with NDD (S.B.-F.) Authorization was requested from those responsible for each entity. Once authorization was obtained, the interested parents were contacted, and the questionnaire was provided in digital format. The participation of all parents was voluntary. Before starting to fill in the questionnaire, the objective of the study was explained to them, and their written informed consent was requested. The researchers resolved the doubts that arose to the parents while completing the questionnaire. Similar to the original questionnaire in the English version, the responses were based on parents’ perceptions of their children’s play. The same procedure was followed in all centers.

The inclusion criteria in the neurotypical group were to be the main caregiver of a child aged between 3 and 9 years old without any neurological or psychiatric illness, without learning disorders, Spanish nationality, and consent to participate in the study. The criteria in neurodevelopmental disorder group in addition to age were to have a clinical diagnosis of NDD (ASD, DD, or SLD).

### 2.2. Instruments

The My Child’s Play (MCP) is the original English version of the questionnaire that has been translated and validated into Spanish in this study, and it is a tool that has strong psychometric qualities, with good validity and reliability of internal consistency (Cronbach’s α = 0.86) [26]. The original tool consisted of 50 items, the last version reduced the number of items to 43, being divided into four dimensions: interpersonal relationships and social participation, executive functions, choices and preferences in play, and opportunities in the environment. Parents should indicate in each item the response that best reflects their child’s behavior during play using a 5-point Likert scale ranging from 1 = never to 5 = always. It also includes the option of not applicable to assess an item as not relevant in that specific case. The total score obtained in the questionnaire is interpreted taking into account that higher scores reflect better performance.

The participants answered a series of questions about sociodemographic aspects that included age, gender, place of residence, educational level, and general questions about their children such as premature or full-term birth. Likewise, the parents were asked if the children had any type of learning difficulty and if they did other activities regularly in addition to school, such as sports, playing an instrument, or another hobby. Subsequently, all parents completed the translated version of the MCP questionnaire.

### 2.3. Design and Procedure

For the development of this study, the methodology of the validation of health questionnaires was followed [27]. Before conducting the study, the authors of the original questionnaire were contacted to inform them about our study and request their authorization to carry out the translation, adaptation, and validation of the questionnaire with the Spanish population. This study was approved by the Ethics and Research Committee of the University of Granada (code 1426/CEIH/2020).

The first phase included the direct and inverse translation of the Spanish version and the cultural adaptation that was generated. In the second phase, a pilot study of the Spanish version of the questionnaire was carried out with participants recruited by the research team. The selection of the sample was by non-probabilistic method, and data collection was carried out over six months. Finally, in the third phase, the analysis of the psychometric properties was carried out to check the validity and reliability of the questionnaire in its Spanish version and the confirmatory factor analysis of the structure of the original questionnaire. The original English version of the MCP questionnaire was translated into Spanish by two people independently, a bilingual translator and one of the researchers in the study who has an adequate knowledge of the original language. Both translations were compared simultaneously by the research team to identify and discuss the discrepancies between the two versions until a consensus was reached, generating a first version of the questionnaire in Spanish. The first Spanish version of the questionnaire generated in the previous step was translated back into English by a translator. An expert panel made up of members of the research team was set up to review and compare the translation of each item with the original version and the Spanish version. This allowed us to check whether the translation generated relevant conceptual differences between the version translated into Spanish and the original. A total of five items were modified to improve their understanding, corresponding to numbers 5, 14, 15, 31, and 50 and considering the rest of the questions as correct (Table 1). These changes gave rise to the second version in Spanish of the questionnaire with which the pilot study was carried out.

In a second phase, a pilot test was performed. The Spanish version of MCP was used to carry out a pilot test with 30 parents of children with neurotypical development. An intentional sampling was used. None of the participants reported having problems understanding the instructions of the questionnaire or any of its items. However, one of the participants suggested a possible change toward a more inclusive terminology, modifying the terms “fathers” and “mothers” by parents to also contemplate gay or single parent families. No other changes were made to the wording of the items or instructions of the translated version of the questionnaire.

Once the final questionnaire was obtained, it was administered to parents of children with neurotypical development and NDD. A total of 591 responses were obtained for total sample, of which 17 did not meet any of the established inclusion criteria, leaving 574 valid responses from parents living in Spain. To complete the questionnaire, parents were asked to indicate the answer that best described their children’s play on a 5-point Likert scale from 1 = never to 5 = always. The same instructions were followed as in the original questionnaire. Parents completed the questionnaire at home. In case any questions arose while completing the questionnaire, they were provided with the email of the main researcher and the telephone number of the contact researcher. All parents who agreed to participate in the study completed the questionnaire. Once the questionnaire was completed, it was reviewed by the group of researchers, and in case of doubts or observations made by the parents, they were contacted to resolve possible doubts or suggestions.

### 2.4. Statistical Analysis

Construct validity allows knowing the degree to which the items of the instrument measure the theoretical construct that they intend to measure. The construct validity of the questionnaire was determined through factor analysis, which is a statistical technique that allows structuring a dataset into factors or components. AMOS extension (version 18.0) is structural equation modeling software that was used to conduct confirmatory factor analysis (CFA). The maximum likelihood method was used for the estimation of the goodness of fit parameters. Once it was verified that the goodness of fit parameters did not allow confirming the model with Pearson’s correlations and neither did they improve after eliminating with less weight in each factor, a new CFA was carried out with the FACTOR software (version 10.10.03) [28], since the answer options of the questionnaire are through a Likert scale with 5 answer options. Polychoric correlations with robust analysis and unweighted least squares (ULS) were used, and the method for factor extraction was normalized varimax [29,30]. CFA was performed to verify that the dimensions identified by the authors of the original tool were valid in the translated version of the questionnaire. For this, the measures of the quality of the fit of the model were evaluated through the absolute fit measures: the Chi-square divided by degrees of freedom (CMIN/DF), the Root Mean Square Error of Approximation (RMSEA), incremental adjustment measures such as the comparative fit index (CFI), Tucker–Lewis index (TLI), and other adjusted goodness fit statistics: goodness of fit index (GFI), adjusted goodness of fit index (AGFI).

Reliability of internal consistency of the MCP questionnaire was calculated using Cronbach’s alpha. Reliability for each factor was carried out by calculating this coefficient for the items with respect to the global score and the other coefficient for the items of each domain with respect to its value. Cronbach’s alpha values >0.70 were considered acceptable to guarantee the internal consistency of the questionnaire [31].

Furthermore, relative operating characteristic (ROC) analysis was performed to know the MCP’s ability to classify between children with NDD and neurotypical development. The characteristics of the participants were analyzed using simple descriptive statistics. These statistical analyses were performed using IBM SPSS Statistics for Windows software (version 26.0, IBM Corp., Armonk, NY, USA), for others statistical analysis of the data. Statistical significance was set at *p* < 0.05 (bilateral).

## 3. Results

The sample in this study was 574 families of children (326 boys, 56.8%, and 248 girls, 43.2%) between 3 and 9 years old. Of these, 469 (81.7%) were children with neurotypical development and 105 (18.3%) were children with NDD: DD (*n* = 47; 8.2%), SLD (*n* = 23; 4%), and ASD (*n* = 34; 5.9%). The mean age of the children was 5.55 years (SD = 1.92), and the mean age of the parents was 39.41 (SD = 5.36) and 39.38 (5.23) respectively. Regarding the characteristics of the children, birth was premature in 59 cases (10.3%) and at term in 515 (89.7%).

### 3.1. Construct Validity

**Figure 1:**
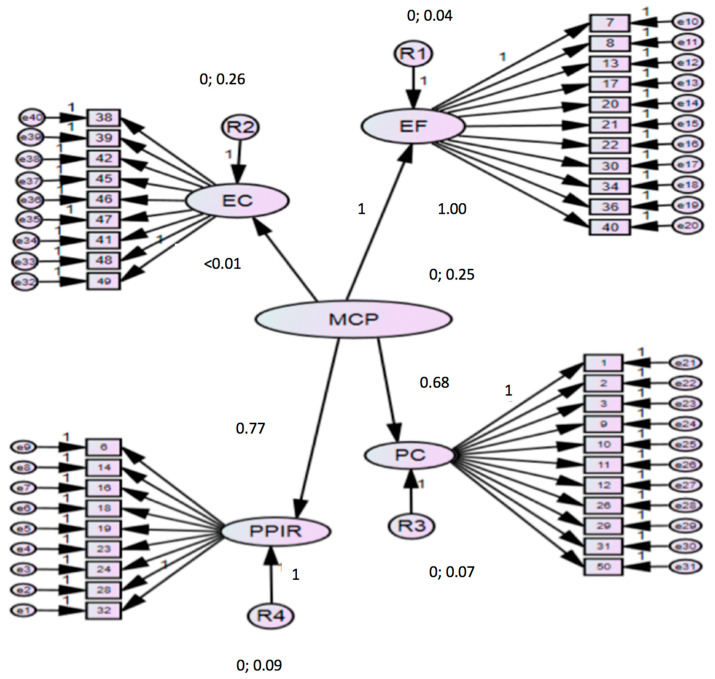
Model for neurotypical, specific language disorder (SLD), and autism spectrum disorder (ASD) children. χ2 = 817.584, gl = 737.89 *p* = 0.020.

The construct validity was verified using a CFA with the factor model proposed by the authors of the original questionnaire conforming to the data that we have obtained in the Spanish population (Figure 1). The CFA with FACTOR Software confirmed the four-factor structure for the MCP (Table 2).

The composition of the four factors and their factor loading are shown in Table 3. The four MCP factors explain 42.98% of the variance. The first factor, flexibility and executive attention, explained 25.40% of the variance. The second factor, the environmental context, explained 7.11% of the variance. The third factor, play characteristics, explained 5.69%, and the last factor, play preferences and interpersonal relationships, explained 4.77%. Items 27, 15, and 35 were not included in any factor.

The final questionnaire in the Spanish version consisted of 40 items instead of 43 in the original version (Table 4 & Appendix A). 

### 3.2. Reliability

The reliability analysis showed a Cronbach’s alpha for total score (0.695) that is acceptable. Despite being slightly below the level of acceptability that was set, we can assume that it is practically 0.7, and therefore, it is an acceptable value, indicating that the internal consistency is adequate (Table 3).

### 3.3. Interpretability

**Figure 2:**
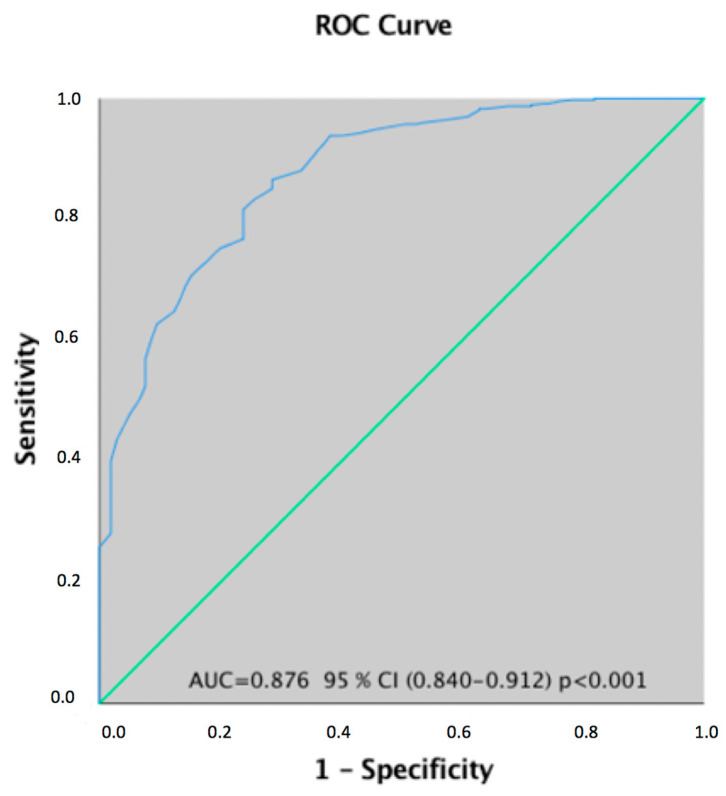
Relative operating characteristic (ROC) curve used to determine the predictive value of MCP in the diagnosis of children with neurodevelopmental disorder.

Table 5 shows the mean scores obtained by neurotypical children and by those with NDD in MCP and in each of its four factors. There were significant differences between neurotypical children and children with NDD with lower scores in the first group (Table 5). Likewise, Figure 2 shows the ROC curve for the predictive level of MCP in the children with NDD. The area under the curve was 0.876 (CI 95%, 0.840–0.912).

Accordingly, the optimal cut-off score to differentiate children from the neurotypical group versus children with NDD was 142 points. Thus, the score <142 in the MCP is indicative of NDD according to the MCP.

## 4. Discussion

The objective of this study was to translate and culturally adapt the MCP questionnaire to the Spanish population, in addition to studying its reliability and validity, providing a new assessment resource for pediatric health and educational professionals that allows them to learn about participation in play in children with neurotypical development and children with NDD. Play is of great importance and very significant in childhood, the assessment of which has been hampered by the complexity for defining the concept and because it is a behavior that is difficult to standardize and quantify. For professionals and those interested in assessment, the lack of time and resources and having only clinical environments where the behavior during play is different from that manifested in the family environment means that many professionals do not use the existing standardized tools [32,33]. These problems can be addressed with questionnaires such as My Child’s Play. Scores on the MCP questionnaire represent the parental perceptions on daily living. Although MCP does not include observations or tests of the child’s play performance, it could also be considered a strength, since it is relevant from the point of view of family-centred practice, allowing to know the functioning of the child in everyday life and showing good ecological validity. MCP has good psychometric properties [25]. The MCP allows knowing different factors underlying the play that are relevant in the assessment of children with neurodevelopmental disorders: (1) cognitive such as executive functioning; (2) socio-emotional, such as social interaction and participation; (3) the behavior during play; and (4) characteristic of play and child’s play preference. Finally, the MCP is a short questionnaire, easily understandable by parents, and easy to complete.

### 4.1. Reliability

The questionnaire shows good reliability. Cronbach’s alpha coefficient indicates that the internal consistency is acceptable (α = 0.695 for the total score, while in the original, it is 0.86). In addition, in the four dimensions Cronbach’s alpha were 0.861, 0.639, 0.838, and 0.821, respectively. These values were similar to those of the original questionnaire, which were 0.80, 0.81, 0.67, and 0.63. As with the original questionnaire, the factor referring to the environmental context was the one that presented lower reliability.

### 4.2. Construct Validity

Regarding the construct validity, the results obtained in the CFA of the Spanish translation of the questionnaire do not allow us to support the same original factorial solution of the English version. However, both versions have four factors, although the weight of each of them is different for the two populations. The factor analysis of the original questionnaire revealed the existence of four factors (executive functions, interpersonal relationships and social participation, preferences and choices in the game, and opportunities in the environment) that explained 30% of the total variance and supported the original concept of the authors of the elaboration of a questionnaire based on the person–occupation–environment relationship. The CFA of the version translated into Spanish determines that the construct validity is not completely adequate, as indicated by the values produced by the adjustment variables (RMSEA = 0.058, CFI = 0.792, NNFI = 0.780, and CIM/DF = 2.937); therefore, the model proposed in the original questionnaire did not fit the data obtained in the Spanish population. However, the new factorial solution obtained also supports the person–environment–participation relationship proposed by the original authors. It is important to note that both populations can identify the same factors, although in a different order, and that therefore, the overall structure of the questionnaire is similar in both versions. In the Spanish version, the first factor was executive functions, the second factor was the environmental context, the third factor referred to the characteristics of the play, and the fourth factor can be understood as referring to preferences in the play and interpersonal relationships.

The differences between the original model and the Spanish version may be due to different factors. Probably the most important is that the sample used in our study included children not only with neurotypical development but also with NDD according to the prevalence in the Spanish population [34]. This allows us to differentiate between children with neurotypical development and NDD through the MCP, as indicated by the results of the ROC curve.

This explains the fact that the first factor, referring to executive functions, refers fundamentally to cognitive flexibility and self-regulation of attention, which can be a weak point in children with ASD and SLD. In addition, these functions keep changing between 3 and 9 years [35,36], so it is relevant that it emerges as an important factor in the child’s play. Executive functions develop during childhood and are complex mental activities that allow the child to plan, make decisions, show a flexible behavior, to change from one task to another, or have inhibitory control during play. In addition, the play contributes to the self-controlled development of executive functions, obtaining greater benefits when it is slightly structured [37]. Furthermore, the development of executive functions has been related to parenting patterns. In this way, it is possible that these differences are showing different customs and traditions than in the way in which parents understand the play, the type of context in which it is played, the rules, and demands of the context. These aspects are coherent if one takes into account that the play, as a human occupation, has a social and anthropological component and not merely a cognitive one [38].

Another possible explanation for the differences found is that, in the original research, only mothers were included, compared to the inclusion of both parents in our study. The relationship of mothers and fathers with their children is different, and many authors have agreed that today, there is still a greater involvement and presence of women in parenting, that leads to a greater appearance of positive interactions compared to fathers [39]. Furthermore, the sociocultural context has a fundamental role in explaining the play [40].

The Spanish version consisted of 40 items. Removed items from the updated version of the MCP were the following: 27 (My child persists at play only with toys/games that he likes and finds interesting), 15 (My child is able to cope with frustrating situations that arise during play), and 35 (My child prefers to play for long periods with toys and materials that enable him to touch different textures). The first and second items can be related to both executive functions and self-regulation of attention and can be related to items 7, 22, and even item 10. On the other hand, the elimination of item 35 may be due to this type of play in Spanish culture being considered suitable between 18 and 36 months [41,42].

### 4.3. Interpretability

This study provides preliminary evidence of the discriminant validity of the MCP among children with neurotypical development and NDD, as shown by the scores for factors 1, 3, and 4. In addition, the total MCP score allows for the consistent differentiation of children with disorders of NDD with neurotypical development, with the established cut-off point of 142.

### 4.4. Implications in the Practice

This study is especially relevant since it is the first one to provide a version in Spanish for the assessment of the play. It provides a simple and quick tool for the evaluation of the play by health professionals and educators, allowing the detection not only whether there are differences between the play of children with NDD or neurotypical development, but it also allows us to know the dimensions in which there are differences, such as executive functions, play preferences, and interpersonal interactions, environmental context, and/or play characteristics. The MCP has a series of advantages for the assessment of play in childhood, allowing guiding treatment on the underlying factors that affect successful participation in the play.

In addition, it is a short, simple, and easy questionnaire for parents to fill out that allows for relatively easy screening. This questionnaire can facilitate professionals working in the field of childhood, to establish intervention programs and treatment plans in children between 3 and 9 years, and with NDD, especially in those with ASD, SLD, and DD.

### 4.5. Limitations and Future Research

The present study has some limitations. First, we took a non-random sample. Therefore, the study should be replicated in a representative random sample of parents of neurotypical children. Second, it would be advisable to expand the sample size further and be able to establish levels according to different age groups. As a future line of research, it would be of interest to compare different clinical populations with other NDD, such as attention deficit hyperactivity disorders or motor coordination disorders and to establish different profiles in the play, which allow orienting the targeted intervention for these specific groups. Likewise, it would be of interest to be able to develop instruments for younger children and babies not only based on parental perceptions but also on the child’s performance in the play.

## 5. Conclusions

The MCP provides a unique understanding of the processes underlying the play; especially, it allows knowing how some executive functions influence during it, such as cognitive flexibility and attentional control. The MCP allows us to assess the play from a broad perspective. In this sense, it helps us knowing which elements of the child’s participation are appropriate to the context, as well as whether the conditions of possibility allow the child to participate positively in the play. It also gives the opportunity of knowing the characteristics of the child’s play, their preferences, and social interaction during it.

The results of cross-cultural validation and psychometric analysis confirm its internal consistency, as well as the construct validity and discriminant validity in the Spanish population and with children with ASD, SLD, and delayed development.

## Figures and Tables

**Table 1:** Items modified by experts.

Item	First Spanish Version	Second Spanish Version
5	El niño es capaz de imitar movimientos.	El niño imita movimientos.
14	El niño se adapta fácilmente a la intervención de nuevos adultos o niños.	El niño se adapta fácilmente a nuevos adultos o niños.
15	El niño es capaz de afrontar situaciones de frustración durante el juego.	El niño afronta situaciones de frustración durante el juego.
31	El niño pierde el interés cuando juega por sí mismo.	El niño pierde el interés cuando juega solo.
50	Estoy contento con la forma en que mi hijo juega.	Estoy satisfecho con la manera con la que juega el niño.

**Table 2:** My Children’s Play goodness-of-fit index from the confirmatory factor analysis.

	Index	Cut-Off	Original Model Value	Alternative Model Value
Model Fit				
	p (χ2)	>0.05	2161.378 *p* < 0.001	817.584 *p* = 0.020
	RMSEA	<0.05	0.058	0.023 CI 95%, (0.010–0.050)
Incremental Adjusted Measures				
	CFI	>0.90–1	0.792	0.991
	NNFI	>0.90–1	0.780	0.990
	CMIN/DF	<2	2.937	1.108
	RMSR	<0.08	-	0.0479

p (χ2): Chi-squared probability; RMSEA: root mean square error of approximation; CFI: comparative fit index; NNFI: non-normed fit index, RMSR: root mean square of residuals.

**Table 3:** Factor loading for the 40 items of the My Child’s Play questionnaire.

Item No.	Item	Factor 1	Factor 2	Factor 3	Factor 4	
Executive Functions: Flexibility and Executive Attention						
7	Child has difficulty concentrating with background noise	0.381				Eigenvalue: 10.92 Cronbach’s alpha: 0.861 IC95% (0.844–0.878)
8	Child bumps into or drops things during play	0.650				
13	Child adapts easily to changes in play conditions	0.572				
17	Child plays with kids according to the rules	0.619				
20	Child is willing to share toys with others	0.420				
21	Child adapts play behavior to setting	0.503				
22	Child controls impulses during play with others	0.693				
30	Child needs lots of breaks to stay attentive	0.557				
34	Child doesn’t play games that have rules	0.538				
36	Child purposely bumps into objects or surfaces	0.583				
40	Child has difficulty playing with too many visual stimuli	0.476				
Environmental Context						
38	There is accessible space outside house for play		0.347			Eigenvalue: 3.058 Cronbach’s alpha: 0.639 IC95% (0.593–0.682)
39	There is accessible space inside house for play		0.446			
41	Child has enough toys for varied enjoyable play		0.468			
42	Toys at home are organized for easy access		0.364			
45	I consider my child’s play preferences		0.331			
46	I offer help after my child tries playing alone		0.614			
47	I model play according to my child’s abilities		0.639			
48	I define rules clearly so my child can play enjoyably		0.521			
49	Daily routine includes time for play with the child		0.568			
Play Characteristics						
1	Child plays with toys according to intended use			0.352		Eigenvalue: 2.44 Cronbach’s alpha: 0.838 IC95% (0.818–0.857)
2	Child varies play with toys			0.458		
3	Child loses interest in toy			0.574		
9	Child persists at play even when having difficulty			0.404		
10	Child tries to problem solve by him- or herself during play			0.521		
11	Child can’t get organized for play without adult help			0.525		
12	Child needs adult help to stay focused on play			0.695		
26	Child finds opportunity to play everywhere			0.561		
29	Child enjoys imaginative play			0.581		
31	Child loses interest when playing by him- or herself			0.663		
50	I’m pleased with the way my child plays			0.601		
Play Preferences and Interpersonal Relationships						
6	Child uses both hands to play				0.322	Eigenvalue: 2.05 Cronbach’s alpha: 0.821 IC95% (0.798–0.842)
14	Child adapts easily to new adults or children				0.687	
16	Child relates to other children during play				0.819	
18	Child is able to initiate play				0.612	
19	Child takes on role of group leader during play				0.522	
23	Child needs adult help to join group of children playing				0.696	
24	Child prefers to play only with familiar toys				0.570	
28	Child avoids play that requires movement				0.488	
32	Child prefers to play with adults over children				0.447	

**Table 4:** Distribution of items for each factor in original and for Spanish version of My Children’s Play.

Factor Original Version	Items Original Version (43 Items)	Factor Spanish Version	Items Spanish Version (40 Items)
Factor 1. Interpersonal Relationships, Social Participation	10, 14, 15, 16,17, 18, 19, 20, 21, 23, 24	Factor 4. Play Preferences and Interpersonal Relationships	6, 14, 16, 18, 19, 23, 24, 28, 32
Factor 2. Executive Functions	3, 7, 8, 11, 12, 22, 27, 30, 31, 32, 34, 36	Factor 1. Executive Functions: Flexibility and Executive attention	7, 8, 13, 17, 20, 21, 22, 30, 34, 36, 40
Factor 3. Play Characteristics and Behavior	1, 9, 13, 26, 28, 29, 35,38, 39, 40, 41	Factor 3. Play Characteristics	1, 2, 3, 9, 10, 11, 12, 26, 29, 31, 50
Factor 4. Environmental Context	2, 6, 42, 45, 46, 47, 48, 49, 50	Factor 2. Environmental Context	38, 39, 41, 42, 45, 46, 47, 48, 49

**Table 5:** Mean scores in neurotypical and neurodevelopmental children.

	Neurotypical Group		Neurodevelopmental Group			
	Mean	SD	Mean	SD	Dif Mean	Cohen’s d
Flexibility and executive attention	43.06	5.63	32.12	7.24	10.94	1.837
Environmental context	38.35	4.12	38.26	4.10	0.089	0.057
Play characteristics	41.21	5.50	34.55	7.56	6.66	1.123
Play preferences and interpersonal relationships	36.10	4.06	28.55	5.50	7.55	1.733
Total Score	158.75	13.50	133.49	17.31	25.25	1.770

## Data Availability

The data presented in this study are available on request from the corresponding author. The data are not publicly available due to privacy reasons.

## Appendix A. Spanish Version of My Child’s Play (MCP) of © Schneider & Rosenblum 2014

El niño juego con los juguetes según el uso previsto.
El niño varía el tipo de juego con los juguetes.
El niño pierde el interés por los juguetes.
El niño utiliza ambas manos para jugar.
El niño tiene dificultad para concentrarse con ruido de fondo.
El niño se choca contra los objetos o los deja caer durante el juego.
El niño persiste en el juego incluso cuando tiene dificultades.
El niño intenta resolver los problemas por sí mismo durante el juego.
El niño no puede organizarse para jugar sin la ayuda de un adulto.
El niño necesita ayuda de un adulto para concentrarse en el juego.
El niño se adapta fácilmente a los cambios en las condiciones de juego.
El niño se adapta fácilmente a la intervención en el juego de nuevos adultos o niños.
El niño se relaciona con otros niños durante el juego.
El niño juega con los demás niños de acuerdo con las normas del juego.
El niño puede iniciar el juego.
El niño asume el papel de líder del grupo durante el juego.
El niño está dispuesto a compartir juguetes con otros.
El niño adapta su comportamiento durante el juego al contexto.
El niño controla sus impulsos durante el juego con los demás.
El niño necesita ayuda de un adulto para unirse al juego de un grupo de niños.
El niño prefiere jugar solo con juguetes familiares.
El niño encuentra la oportunidad para jugar en cualquier lugar.
El niño evita el juego que requiere movimiento.
El niño disfruta del juego imaginativo.
El niño necesita muchos descansos para mantenerse atento.
El niño pierde el interés cuando juega solo.
El niño prefiere jugar con adultos en vez de niños.
El niño no juega a juegos que tienen reglas.
El niño se choca contra objetos y superficies a propósito.
Hay espacio accesible para el juego fuera de casa.
Hay espacio accesible dentro de casa para jugar.
El niño tiene dificultades cuando juega con demasiados estímulos visuales.
El niño cuenta con suficientes juguetes para disfrutar de un juego variado.
Los juguetes están organizados en casa para que sean fácilmente accesibles.
Tengo en cuenta las preferencias de juego de mi hijo.
Le ofrezco ayuda después de que el niño haya intentado jugar solo.
Adapto el juego a las capacidades del niño.
Defino claramente las reglas para que el niño pueda divertirse.
Mi rutina diaria incluye tiempo para jugar con el niño.
Estoy satisfecho en la forma que mi hijo juega.
